# Processing of Multi-dimensional Sensorimotor Information in the Spinal and Cerebellar Neuronal Circuitry: A New Hypothesis

**DOI:** 10.1371/journal.pcbi.1002979

**Published:** 2013-03-14

**Authors:** Anton Spanne, Henrik Jörntell

**Affiliations:** Neural basis for Sensorimotor Control, BMC F10, Lund University, SE-22184 Lund, Sweden; University College London, United Kingdom

## Abstract

Why are sensory signals and motor command signals combined in the neurons of origin of the spinocerebellar pathways and why are the granule cells that receive this input thresholded with respect to their spike output? In this paper, we synthesize a number of findings into a new hypothesis for how the spinocerebellar systems and the cerebellar cortex can interact to support coordination of our multi-segmented limbs and bodies. A central idea is that recombination of the signals available to the spinocerebellar neurons can be used to approximate a wide array of functions including the spatial and temporal dependencies between limb segments, i.e. information that is necessary in order to achieve coordination. We find that random recombination of sensory and motor signals is not a good strategy since, surprisingly, the number of granule cells severely limits the number of recombinations that can be represented within the cerebellum. Instead, we propose that the spinal circuitry provides useful recombinations, which can be described as linear projections through aspects of the multi-dimensional sensorimotor input space. Granule cells, potentially with the aid of differentiated thresholding from Golgi cells, enhance the utility of these projections by allowing the Purkinje cell to establish piecewise-linear approximations of non-linear functions. Our hypothesis provides a novel view on the function of the spinal circuitry and cerebellar granule layer, illustrating how the coordinating functions of the cerebellum can be crucially supported by the recombinations performed by the neurons of the spinocerebellar systems.

## Author Summary

The movement control of the brain excels in the seamless coordination of our multi-segmented limbs and bodies and in this respect the brain widely outperforms the most advanced technical systems. So far, however, there is little knowledge about the neuronal circuitry mechanisms by which this coordination could be achieved. The present paper makes a synthesis of some recent findings of cerebellar neuronal circuitry functions and spinocerebellar systems to introduce a novel hypothesis of how the cerebellar and spinal cord neuronal networks together establish signals that form a basis for coordination control in the mammalian central nervous system. The hypothesis takes into account some recent, surprising findings about cerebellar granule cell function and explains some long-standing enigmas concerning the structure of and information mediated by the spinocerebellar pathways. It describes some interesting parallels between the spinocerebellar network and Artificial Neural Networks (ANNs), and capitalizes on some of the major conclusions from ANN studies to explain the biological observations.

## Introduction

**Figure 1:**
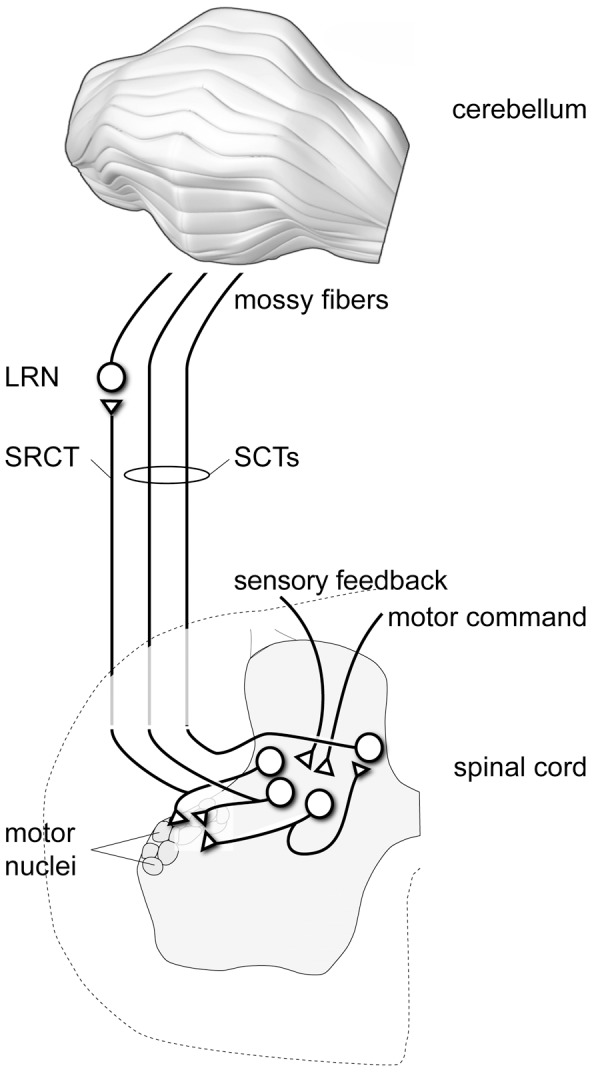
The information provided by the spinocerebellar and spinoreticulocerebellar mossy fibers derives from the spinal interneuron circuitry. The vermis and pars intermedia of the cerebellum receives a substantial part of their mossy fiber inputs from the SCT/SRCT pathways [19]. The SCT/SRCT pathways consist of spinal interneuron projections either directly as mossy fibers (rostral spinocerebellar tract, RSCT), via a relay in the lateral reticular nucleus of the brainstem (spino-reticulo cerebellar path, SRCT), or via relay cells located in the spinal cord (ventral and dorsal spinocerebellar tracts, VSCT and DSCT) [19]. These spinal interneurons can project directly to alpha-motorneurons and likely form an integral part of the spinal motor circuitry, by conveying sensory feedback and motor commands to the motor nuclei of the spinal cord [99].

The coordination of our multi-segmented bodies and limbs is an unsolved computational challenge that is dealt with seamlessly by the neuronal circuitries of our brains. Within the brain, the cerebellum is considered the most important structure for coordination [1], [2] but we know very little about the mechanisms that could underlie this aspect of cerebellar function. The spinocerebellar and spino-reticulo-cerebellar systems are major sources of mossy fiber (MF) inputs for the widespread cerebellar regions with direct connections to the motor systems, the corticospinal, rubrospinal, reticulospinal, tectospinal and/or vestibulospinal tracts [3]–[13] and the corresponding regions of the cerebellum are implicated in limb coordination in man [13]. The neurons of origin of these systems are located in the spinal cord, where they act as components of the spinal motor circuitry (Figure 1) or receive input directly from such neurons. These systems comprise the spinocerebellar tracts (SCTs), i.e. the ventral spinocerebellar tract (VSCT) and its components including the spinal border cells (SBCs), the components of the dorsal spinocerebellar tract (DSCT), the rostral spinocerebellar tract (RSCT) as well as the spino-reticulocerebellar tracts (SRCTs) providing MFs to the cerebellum via the lateral reticular nucleus (LRN) [14]–[18] (Fig. 1). All of these systems receive sensory feedback either directly or mediated via spinal interneuron systems. They also receive input from supraspinal motor centers, again either directly or mediated via spinal interneuron systems. Single SCT and SRCT neurons can vary with respect to the relative weights of the sensory feedback and motor command components. Considering the influence from motor command systems, it is reasonable to assume that the MFs of these systems carry information to the cerebellum regarding on-going movements, rather than transmitting information resulting from passive sensory stimulation [19]. However, why this combination of different sensory and motor signals occurs before the level of the cerebellum and how it is used by the cerebellum is not well understood.

A second issue of cerebellar function is the tonic inhibition of granule cells (GrCs) in the mature mammalian cerebellum. The inhibition of GrCs in the adult cerebellar cortex, supposedly mostly due to Golgi cell release of GABA, primarily consists in tonic and slowly modulated inhibition whereas fast inhibitory postsynaptic potentials are weak or absent [20], [21]. A couple of observations indicate that GrCs are designed to be tonically inhibited. The tonic GrC inhibition is to a large extent mediated via alpha-6-containing GABA(A) receptors [22], [23], which is a type of receptor that is characterized by long-lasting inhibitory effects and is present at uniquely high concentrations in the cerebellar granule layer [24]–[26]. The second observation is that even when the alpha-6 subunit is knocked-out using genetic engineering, GrCs seem to compensate for this loss of hyperpolarisation by increasing the expression of potassium conductances [27]. At the same time as the tonic component of the GrC inhibition develops, the traditional fast inhibitory response is gradually lost [20], [28]. Accordingly, GrC responding with burst responses to skin stimulation show little sign of Golgi cell inhibition, even though the afferent Golgi cells are relatively strongly activated by the same stimulation [21]. However, although the contribution to the immediate, fast information processing may be modest, Golgi cells can still contribute to cerebellar processing by regulating the difference between the resting membrane potential and spike firing threshold of the GrCs by modulating the level of tonic inhibition. This arrangement could result in that the GrC resting membrane potentials are distributed across the population of cells. Consistent with this idea, the resting membrane potentials of GrCs *in vivo* [29] had a mean value of −64 mV, but reached at least as low as −80 mV and as high as −40 mV.

A third conspicuous observation is that for GrCs receiving cutaneous input, the 3–5 MF synapses that the GrC receives have been reported to carry functionally equivalent inputs. In a study focused on the cuneocerebellar tract [30], in which the individual MFs carry submodality-specific input from small receptive fields primarily from the distal forelimb, the GrCs were found to sample submodality- and receptive field-specific inputs [21], i.e. information that was equivalent to that of the individual MFs. In an extended study, where cutaneous inputs mediated via the SCT and SRCT systems were included, the MF inputs to individual GrCs were found to be similarly coded, i.e. they originated not only from same receptive field and same submodality, they were also found to originate from precerebellar cells that coded for that particular skin input in the same way [31]. This ‘similar coding’ principle of MF to GrC innervation was suggested to be due to the fact that different afferent pathways process the skin afferent input in different ways, with different degrees of involvement of other modalities and/or descending motor commands. In order to preserve the information generated by the afferent systems similarly coded MFs needed to converge on the same GrCs [19]. This view is supported by numerous anatomical studies, showing that different afferent pathways have focal rather than diffuse termination patterns in the cerebellar cortex [6], [32]–[34] and that different afferent pathways have complementary distributions in the GrC layer [35]–[37]. Hence, at least for the cerebellar regions involved in limb control, MFs carrying functionally equivalent inputs preferably target the same set of GrCs. A consequence is that the probability of finding GrCs sampling functionally disparate input would be expected to be low. Altogether, the findings of these studies were at odds with previous theoretical predictions that GrCs were expected to integrate MF information from widely separate, functionally disparate sources [38], [39], and presented a challenge to our understanding of the cerebellar granule layer and thereby of the cerebellar cortex in general.

In the present paper, we seek a theoretical explanation for how these three observations can be understood in terms of the function of the spinal and cerebellar neuronal circuitry and how they could provide for the integration across input dimensions necessary to achieve coordination. In the initial part of the paper, we account for the main settings of our hypothesis to describe how sensorimotor functions can be generated in the SCT and SRCT MF systems and how the cerebellum could integrate this information to approximate useful functions. We continue by exploring how non-linear interactions between input dimensions could be handled in the spinal circuitry and the cerebellum and the limitations that may apply to these systems in this respect. For this purpose we construct a simple static model and use a concrete example of non-linear interactions between input dimensions used in the performance evaluation of previous cerebellar models.

## Models

### Properties of the Purkinje cells and interneurons of the molecular layer

As the Purkinje cells (PCs) constitute the only output from the cerebellar cortex, their responses to stimuli reaching the cerebellum through the MFs determine what the cerebellar cortex is able to do, and what type of transformations of the input signals that are possible. While Albus [38] viewed the PC as a binary perceptron for classification, he also acknowledged that the PC is able to modulate its relatively high spontaneous firing frequency, creating in principle a continuous output signal. The spontaneous firing rate of simple spikes can be modulated in both excitatory and inhibitory directions by specific inputs, in a manner consistent with their generation by excitatory parallel fiber (PF) and inhibitory interneuronal inputs, respectively, and with these inputs being summed primarily in a linear fashion [40]. Since the molecular layer interneurons are innervated by the parallel fibers and in turn inhibit the PC, the weights between PFs and PCs are allowed to become negative [40], [41]. The bidirectional plasticity and the complementary location of the receptive fields in PCs and interneurons confirm this assumption [42], [43]. Eq. (1) describes a simplified relationship between PF and PC activity, and forms the basis of the later mathematical models of this paper, where the properties of the neurons in the granular layer will also be taken into consideration.
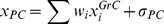
(1)where 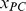 is the PC activity, 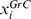 the activity of the 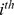 GrC, 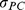 is the spontaneuos activity of the PC and 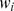 is the synaptic efficacy between the 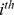 GrC and the PC. Note than 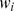 is allowed to be negative since the GrC input can be mediated through inhibitory interneurons [40], [41].

### Properties of the granule layer

A distinct feature of GrCs is the marked difference between the resting membrane potential and the threshold potential for spike firing [21], [29], [44]. Once the spike firing threshold is crossed, however, the input-output relationship of the GrCs is approximately linear [29], [45]. The activity of a GrC can be approximated to be,
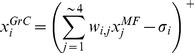
(2)

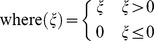


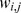
 represents the synaptic weight between the 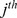 MF and the 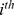 GrC and 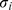 is the distance to the spike firing threshold from the GrC resting membrane potential. The firing threshold is constructed through the use of a ramp function 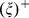, which is defined to be equal to 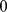 whenever 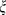 is equal to or lower than 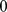, but equal to 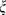 whenever 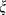 is larger than 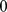. When the synaptic input depolarizes the neuron above 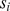, the GrC spike output 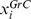 will also start to increase in proportion to the synaptic input. Eqs. (1) & (2) assumes that the MF input to the cerebellum is rate encoded, consistent with existing *in vivo* studies of MFs in awake animals during behaviour [46]–[49], and during fictive locomotion [50]–[52].

As pointed out above, previous observations indicate that an individual GrC sample functionally equivalent input from all of its incoming MFs [21], [31]. As individual MFs branch to contact up to thousands of GrCs [33], the GrC layer would be expected to contain a highly redundant representation of MF inputs, which is also suggested by a systematic investigation of MF receptive fields [53]. Due to neural noise and the limitations of spike encoded transmission of information [54], some redundancy in the GrC representation would be expected to be needed to average out the noise. The very large number of GrCs innervated by the same MF does however suggest that the GrC population by some means recode the incoming MF signals, rather than just compensate for the inherent noise in the afferent signal. In the following section, we propose a solution for how useful expansion recoding can be performed by a redundant population that sample functionally equivalent input. In this view, the role of the Golgi cell to GrC tonic inhibition, potentially supplemented by other sources of GABA input as described in the discussion, is to allow the GrC responses within a redundant population to become sufficiently varied, hence playing a crucial role of the proposed expansion recoding.

### Populations of redundant GrCs as piecewise-linear approximations

**Figure 2:**
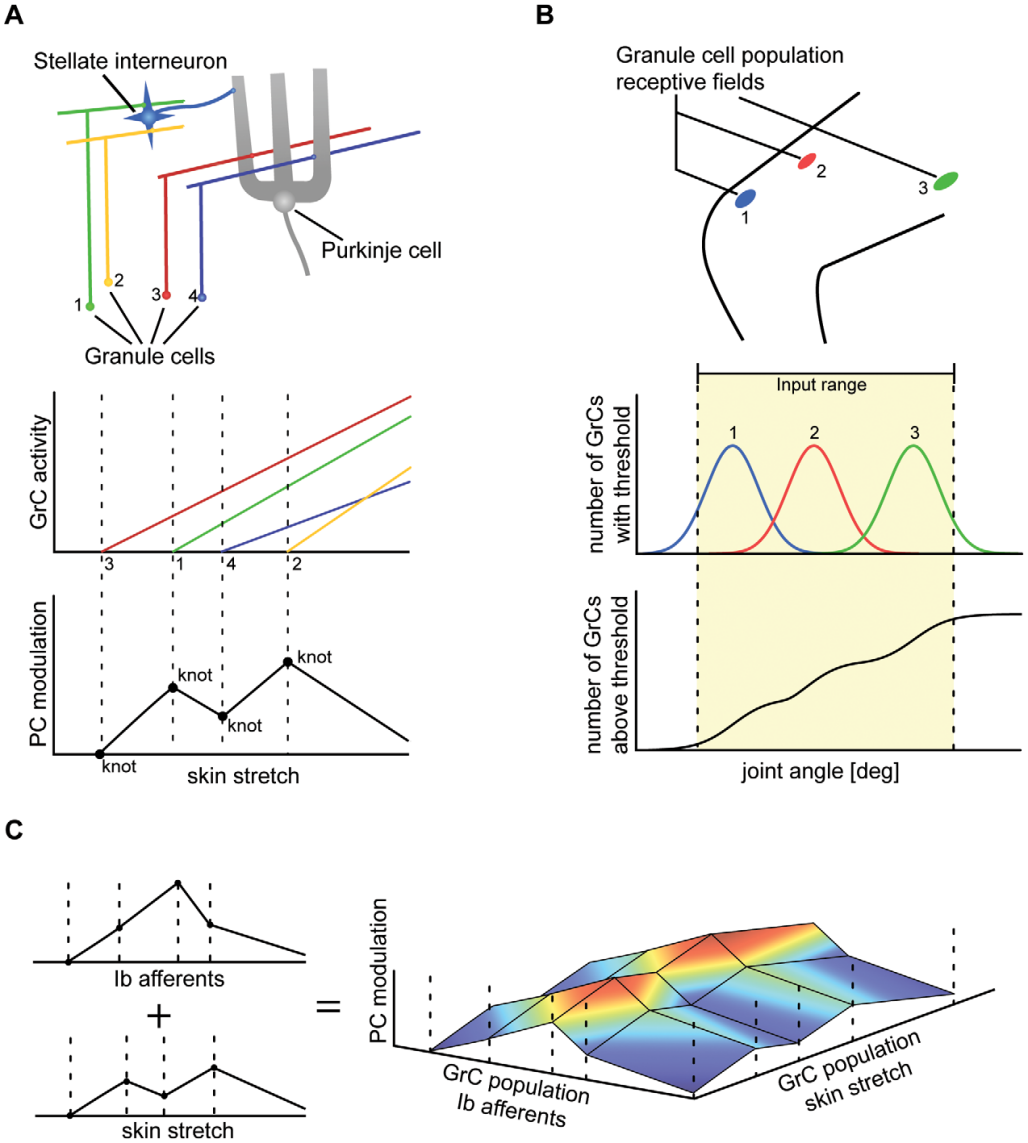
Piecewise-linear (PL) approximations in the cerebellar neuronal network. (A) Using the excitatory input directly from PF and the inhibitory pathway through molecular layer interneurons, the PC can construct a PL approximation of arbitrary non-linear functions of the input reaching the GrCs. (top) Two PFs innervate the PC directly (3 & 4), while the other two innervate a stellate interneuron (1 & 2). (middle) The four GrCs have slightly different thresholds and varying MF efficacy leading to varying activity slopes. (bottom) The PC modulates its output using the input coming from the GrCs according to Eq. (1). The path through the inhibitory molecular layer interneurons allows the weight and thus the slope of the curve to be negative. Each GrC threshold corresponds to one knot in the PL PC output. (B) The distribution of GrC thresholds over the input range determines how well the PC can approximate the non-linear regions of the approximated function. (top) Several receptive fields can contribute to measure a single intrinsic dimension. In this case, the skin stretch can be used to deduce the joint angle. (middle) The different receptive fields allow the GrC thresholds to be spread over a larger input range than that using only a varying degree of Golgi cell tonic inhibition. (bottom) Sum of activity of all GrCs activated from the three receptive fields. As the population GrC activity rises over the entire input range, their output could be used to approximate non-linearities over the entire input range. (C) A naïve approach to enable the PC output to approximate functions of two-dimensions. In this example, afferent information from skin stretch and Ib afferents are added separately in the PC, generating an approximated surface. At each point in the input space, the PC output is calculated by adding the contribution from GrCs innervated by the two separate afferent types.

Using a population of redundant GrCs, which sample functionally equivalent MF input and differ with respect to the separation between the resting 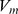 and the spike firing threshold, it becomes possible for the PC to combine the inputs from these GrCs into a piecewise-linear (PL) approximation of an arbitrary smooth function, see Figure 2A. In this case, even if each GrC in the population would receive exactly the same MF inputs, the diversified thresholds in the population of GrCs would be a useful feature for the PC that integrates these signals. Since the GrC thresholds directly correspond to the knots of the PL-approximation (see Figure 2A), the number of GrCs is directly related to the approximation error, which for PL approximations is bounded by 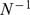, where 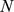 is the number of knots or GrCs. In other words, as new knots are introduced, the upper bound of the approximation error will shrink in proportion to the number of new knots.

### A simple example

To further develop this reasoning, we proceed by considering a simplified example of input from knee skin afferents, for which a relatively straight-forward relationship between knee-joint angle and firing frequency of single afferents on the hairy skin of the thigh has been reported [55]. In this study, it was shown that skin afferents with receptive fields located at different distances from the knee joint all could code for the knee angle but with different intensity. Considering this example, it is conceivable that in addition to the tonic inhibition, GrC populations with related but not completely equivalent inputs could help differentiate the firing thresholds with respect to the knee joint angle over a larger input region. The combined effect of both Golgi cell inhibition and related receptive fields is illustrated in Figure 2B. In accordance with this view, the distribution of the active range, with respect to joint angle within a population of muscle-spindle afferents, has been found to cover the entire range of joint-angle positions investigated, while any individual afferent from the population had a much smaller active range [56]. The properties of the one-dimensional example in Figure 2B do, however, not tell how the one-dimensional arrangement could be expanded to integrate signals related to more than one input dimension and approximate complex, possibly non-linear interactions between them. To approach this issue, we consider in addition to the skin stretch sensors another type of sensor, the Ib afferents, the firing frequency of which is related to muscle force [57]. Note that even though we are presenting these ideas using sensory inputs, the following line of reasoning would also apply to information from the motor command domain, but since the exact content and format of these signals is less well known it would make for a worse example. For example, skin stretch might well be replaced with motor command in Figure 2C below.

The naïve approach to multidimensional input would be to simply superimpose one PL approximation along each dimension, see Figure 2C. Each point in the input space of the combined approximation, would map to the sum of all the separate one-dimensional approximations. Relating this approach to the cerebellar structure, it would correspond to each GrC receiving signals from a single input dimension. The PC, working as a linear integrator of GrC inputs, would then integrate information from the input dimensions available in the GrC population contacting this PC via the parallel fibers, hence integrating the information into multiple combined PL-approximations of these input dimensions. But, a presumed core cerebellar function is to improve coordination, which depends on interactions between adjacent limb segments, between multiple modalities or submodalities and between motor command and sensory feedback, i.e. interactions that are most likely non-linear. It is not obvious to what extent the naïve approach could be used to approximate such non-linear interactions. Given the description of the MF-GrC system so far, it is possible to interpret Eqs. (1) and (2) as a radial basis-function network with PL radial basis functions [58]. Light and Cheney [59] showed that such a network cannot approximate arbitrary multivariate functions to arbitrary precision with no recombination of the input signals prior to the hidden layer (‘hidden layer’ corresponds to a layer of neurons located between the input layer and the output layer of the system, i.e. in our case the GrCs). Consequently, if the cerebellum is to be able to approximate arbitrary functions, it is not enough to have raw afferent signals as MF content as in the naïve approach described above – there is a need for the signals to be recombined.

### Hypothesis - General static model of the spinocerebellar system

**Figure 3:**
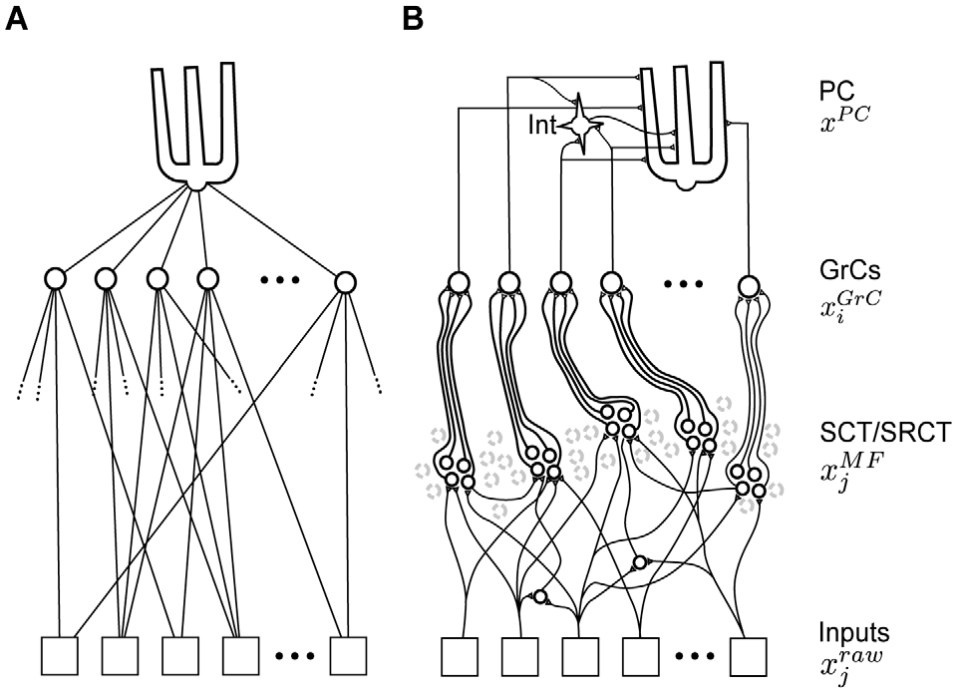
Comparison of network structures of ANNs and spinocerebellar systems. (A) A standard feed-forward ANNs with one hidden layer (GrCs), where every input is available to all units in the hidden layer. (B) In contrast, in the spinocerebellar system, MF inputs to GrCs have a focal termination, where different functional types of MFs are connected to different sets of GrCs. In this arrangement it is possible for recombination of the sensorimotor inputs to take place already at the level of the SCT/SRCT units, while the recombination at the granule layer is restricted to the approximately four functionally similar MFs that innervate every GrC. In the biological system, the GrCs have only excitatory synapses upon the PCs, i.e. only positive weights. It is however possible to obtain inhibitory GrC to PC efficacies by mediating the GrC signal via the inhibitory interneurons of the molecular layer (Int) (cf. [40], [100]).

In contrast to the naïve approach, the most general approach would be to allow each GrC to receive any combination of all the inputs that reach the cerebellum via the MFs as illustrated in Figure 3A. It is possible to formalize such a multidimensional PL approximation into the following equation, in order to investigate its properties:
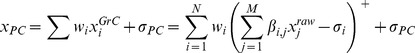
(3)where 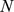 is the number of GrCs and 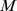 is the number of raw signals transmitted through the MFs. As opposed to in Eq. (2), 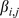 is not a single synaptic efficacy of the MF GrC transmission, but the total transmission efficacy between the 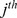 raw input signal and the 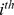 GrC, which includes contributions from one or more MF-to-GrC synapses. We use 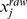 to denote signals that have not been recombined, e.g. sensory signals related to a single input dimension. It should be noted that Eqs. (2) and (3) are not in contrast to each other, since in Eq. (2) the efficacies between the raw signals and the MF activities are not mentioned. In other words, any situation that can be described by Eq. (3) can also be true for Eq. (2) and Eq. (1) by the correct choice of efficacies between raw signals and the MF activities.

Eq. (3) has close links and mathematical similarities to several concepts in multivariate regression and in particular artificial neural nets (ANN), see Bishop [60]. It is a special case of a feed-forward ANN with a single hidden layer, where each hidden unit would correspond to a GrC, and all hidden units have ramp activation functions. In Eq. (3) and Figure 3A, as in a regular feed-forward ANN, each hidden unit (i.e. GrC) is innervated by all units in the input layer (i.e. raw input signals). Marr and Albus assumed such multimodal recombination as an essential part of their models, in order to expand the input onto a higher dimensional space enabling non-linear classification. It is known that in theory such networks can approximate arbitrary functions [61], but in practice they often require either a very large amount of neurons or extremely large synaptic weights to approximate high dimensional or complex function surfaces [62].

### Recombination of inputs - projections in input space

Available evidence from the mammalian cerebellum, however, does not support any substantial multimodal recombination within the granule layer (see Introduction) and the linear integrative and firing properties of the individual GrCs combined with the rate coding in the SCT/SRCT systems (see above) do not seem well suited for calculating non-linear functions. But extensive multimodal recombination of inputs does occur in the major MF pathways, i.e. in the neurons of origin of the SCTs and SRCTs [8], [14]–[17], [49], [63]–[70], and they are a major source of integrated sensorimotor information to the cerebellum. The biological observations consequently suggest an alternative view of the spinocerebellar network structure (Figure 3B). Notably, in this view, the multimodal recombination that is necessary in the Marr-Albus type of classifier is retained but the recombination is placed prior to the granule layer.

The recombination of two or more input dimensions can be viewed as a projection in the input space defined by the available input dimensions. The term projection is used because a recombination of the input variables, provided that it is a linear recombination, can be seen as a projection along a straight line. It should be noted that even though we in the account below primarily discuss linear projections, also non-linear projections are valid. In fact, non-linear recombinations could differentiate the input in addition to the firing thresholds of the GrCs. In specific cases, such non-linear recombinations have been shown to improve the quality of approximations containing non-linear interactions between input dimensions [71]. Since the SCT/SRCT neurons are located within a complex network of spinal interneurons, non-linear functions can potentially be generated here.

If the available recombinations of the input dimensions are present already at the stage of the MFs (Figure 3B), then the number of MFs imposes a restriction on how many projections that can be represented in the system. The number of GrCs is much higher than the number of MFs [33], [40], but as noted above, if the PCs are to be able to make PL approximations along each projection, a population of GrCs is needed to represent each type of sensorimotor combination provided by the MF systems (i.e., to create a large number of knots in the PL approximation) (Figure 2). In any case, as we will see below, the number of GrCs is far too low to be able to accommodate all possible recombinations in the system. Hence, the cerebellum is limited to working with a restricted set of recombinations, or projections. In the case of a restricted number of projections in the available input space, there exist regression models which allow us to further analyze the properties of such a model [72]. Eq. (3) can be changed into the more restricted form
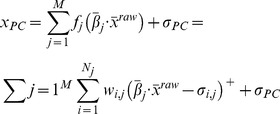
(4)where 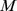 is the number of projections, 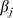 determines the direction of the projection and 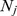 the number of GrCs along projection 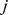. Before we apply the model in Eq. (4) to the double joint arm we will describe to what extent the ‘curse of dimensionality’ [73] affects and probably shapes the properties of the projections available in these systems.

### Limited number of GrCs

To illustrate the curse of dimensionality and its implications for the amount of sensorimotor functions that could be represented in the cerebellum, all inputs to the system is first assumed to be limited to a finite number of values. Since there is a physical limit upon most biological signals, the number of finite values (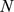) of the input could for example be chosen such that we can achieve a sufficiently good approximation error according to the bound, 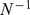, that was presented earlier. The size of the space spanned by one such discretized input could be considered to be 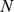. If we introduce another input or dimension to this space, it will become a square with size 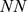. By adding another input dimension, the space will become a cube with size 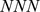.

Hence, the size of the input space grows exponentially with the number of input dimensions. Furthermore, each GrC, through the PF to PC synaptic weight, can only determine the value of the approximation at a single point in the input space [58] (Figures 2,3). If the number of dimensions increase, and the size of the input space grow, the average distance between randomly placed points in that space grows as 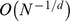, where d is the number of dimensions and N is the number of points spread randomly across the entire space [60]. Hence, as more and more dimensions are introduced to the input space, the number of approximation points or GrCs has to grow exponentially in order to maintain the average distance between these points. Formally, the root mean squared error (RMSE) can be shown to be related to the average distance between points and is bounded by 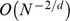 [74]. The exponential growth of the space spanned by all input dimensions and all the resulting implications are what Bellman coined as the curse of dimensionality [73].

In a biological system, one sensory receptor sampling information from one submodality at one locus in the body would correspond to one dimension. In the extreme case, each sensor is considered to sample unique information and therefore represents a unique input dimension. In this case, the total input space would be of astronomical size due to the described exponential growth per dimension. It is completely unfeasible that such a space can be represented to any detail within the brain. To illustrate the exponential growth in numbers even in a simplified system, let us consider a set of receptors where each receptor samples information from one submodality around a single joint. The hand alone has approximately 20 degrees of freedom. In order to encode the entire input space (for simplicity, we only consider static configurations of the hand) with the MF-GrC population in the human (assuming 10 billion GrCs are devoted to hand control, which is likely to be a huge overestimate) the system would be limited to about 3 knots along each input dimension. Hence, even with these crude levels of accuracy in terms of sensory input (and disregarding the role of the motor commands), the number of GrCs needed reaches astronomical figures. If we add the wrist and hence 2 more degrees of freedom to the input, the number of GrCs required for the same crude accuracy would rise to 100 billion GrCs (compared to the estimates of a total of 70–100 billion GrCs in the human cerebellum [75]), illustrating the exponential growth. Again, it is easier to discuss the input dimensions in terms of sensor signals, which we know relatively well what they code for. Nonetheless, it should be recalled that we in addition have to take into account the motor command, which is likely to represent another multi-dimensional set of signals whose functions and interdependencies with the sensor signals would also need to be approximated.

The bounds upon the approximation error we have discussed so far are the worst case scenarios, in which we are assuming that the cerebellum needs to approximate arbitrary functions. As described above, it would require all possible recombinations to be present at level of the granule layer, requiring an enormous amount of GrCs, most of which would remain superfluous if there were no non-linear interactions between the recombined dimensions that actually needed to be approximated. In contrast, if it was sufficient to only use specific projections in the input space, selected to enable the system to approximate specific non-linear functions, the number of MFs and GrCs needed could possibly be substantially reduced. For example, input dimensions that never interact (that we will refer to as ‘superfluous projections’) would not need to converge at all, and it would be possible to approximate additive functions of two or more input dimensions even if they did not converge until the PC layer. Interestingly, Barron [74] showed that it is possible to construct a feed forward ANN that reduces the bound upon the approximation error from 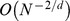 to 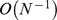, showing that it in principle is possible to reduce the number of needed hidden units in the ANN to the point where the curse of dimensionality is completely avoided but the precision of the approximation is still maintained. While it is unlikely that all requirements to reach the second bound can be fulfilled in the biological system, the results of Barron still demonstrates that finding optimal recombinations of inputs can be used to reduce the number of projections available in the system while maintaining acceptable levels of the approximation errors. Given that it is known that the SCT/SRCT systems represent a limited number (in relative terms) of combinations of sensor and motor signals, this viewpoint has a high biological relevance. Such recombination would correspond to that the SRCT/SCT systems have selected to represent specific projections within the input space. Consequently, the structure of the spinocerebellar network prior to the granule layer needs to be considered in order to explain how the curse of dimensionality can be at least partially lifted.

### Application

To illustrate how the coordination of a multi-segmented limb could work in principle, we consider the simplified example of controlling a planar double joint arm. The planar double joint arm has previously been used to evaluate biophysically detailed models of cerebellar function [76], [77], also including feasible neural delays [78]. Note that the kinematic variables in this example could reside both in the domain of the sensors and in the domain of the motor command – this is not relevant in this example, though, as we only intend to illustrate in principle how projections can be useful in approximating non-linear functions across different dimensions. This example serves the additional purpose of illustrating that a limited number of appropriate projections can go a long way in capturing the interdependencies between different input dimensions. Thereby, this example ties back to the SCT/SRCT systems where more selective, rather than unlimited, combinations of inputs are available. Position, speed and acceleration, i.e. the variables represented in our example, from multiple arm-joints do indeed seem to be represented in individual MFs of the arm-controlling regions of the cerebellum [46] although this does not mean to imply that these are the only variables represented or that the terms in Eq. (5) correspond to the exact interdependencies represented within the spinocerebellar system.

To enable accurate control of the arm, the movement of both joints needs to be coordinated due to interaction torques arising from inertial, centripetal and Coriolis forces [79]. These interactions lead to non-linear terms in the inverse equations of motion of the arm, i.e. the transformation from joint angles, velocities and accelerations to joint torques, which depends on two or three kinematic variables of the arm joints. The three types of non-linear dependencies among the different terms in the inverse dynamics of the planar double joint arm are listed below,
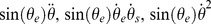
(5)where 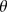 is the joint angle, 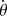 and 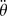 are joint velocities and accelerations, respectively, and the subscripts 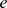 and 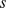 denote the elbow and shoulder joints, respectively. The terms are simplified compared to those in the inverse dynamics [79], but retain all non-linear interactions. In particular, all constant coefficients are removed and all 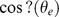 are replaced with 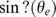 to ease visual comparisons of the results. Variables without subscripts indicate that the term is present within the inverse dynamics with both a shoulder and an elbow variant.

In order to explore how the neuronal circuitry could be used to construct approximations of these equations, we apply the basic static model from Eq. (4) to the non-linear terms in Eq. (5). In particular, we investigate how the input to the cerebellum through the recombination of the input variables influences the quality, or the root mean squared error (RMSE), of the approximations that were constructed.

We also investigated whether selecting particular combinations of input enables better approximations, or if it is sufficient to choose the recombinations at random. By creating approximations of the terms in Eq. (5) with a varied number of random projections as input to the granule layer, it is possible to investigate how reliable an approach using a random selection would be and how many different projections such a method would need to use. To evaluate the results in the case of random projections of input, we compare the approximations using random projections situation with approximations where the directions of the projections were also optimized, instead of chosen at random. Having the optimal projections also allow us to compare approximations using a varied number of GrCs. They can also be used to explore if the optimal projections or at least quality-wise similar (‘good enough’) projections are included in the set of random projections that were used in the previous step. Finally, it also allows us to explore if the results using fewer different recombinations could be significantly improved or compensated by increasing the number of GrCs.

All approximations are constructed by minimizing the error function in Eq. (6), which calculates the RMSE of the approximation compared to the approximated function over a grid of points covering the input space.
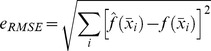
(6)where 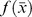 is the approximated function or target function, 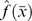 the approximation and the 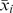 are placed on an equidistant grid covering the input space.

In order to capture the relevant shapes of the functions surfaces of the terms in Eq. (5), the input to the trigonometric functions range between 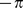 and 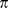. The RMSE is evaluated as the proportion of the maximum RMSE obtained with Eq. (6) having 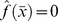. All two-dimensional approximations were constructed using the Levenberg-Marquardt algorithm [80] using the implementation in the Matlab optimization toolbox. Matlabs Nelder-Mead algorithm [81] was found to handle the larger amount of unknowns better than the Levenberg-Marquardt algorithm, due to the need for Jacobians in the Levenberg-Marquardt method, and was used to create the three-dimensional approximations. It is important to note that we use general approximation methods with the intent to prove the possibility to construct approximations with the model rather than to develop a method to construct such approximations.

## Results

**Figure 4:**
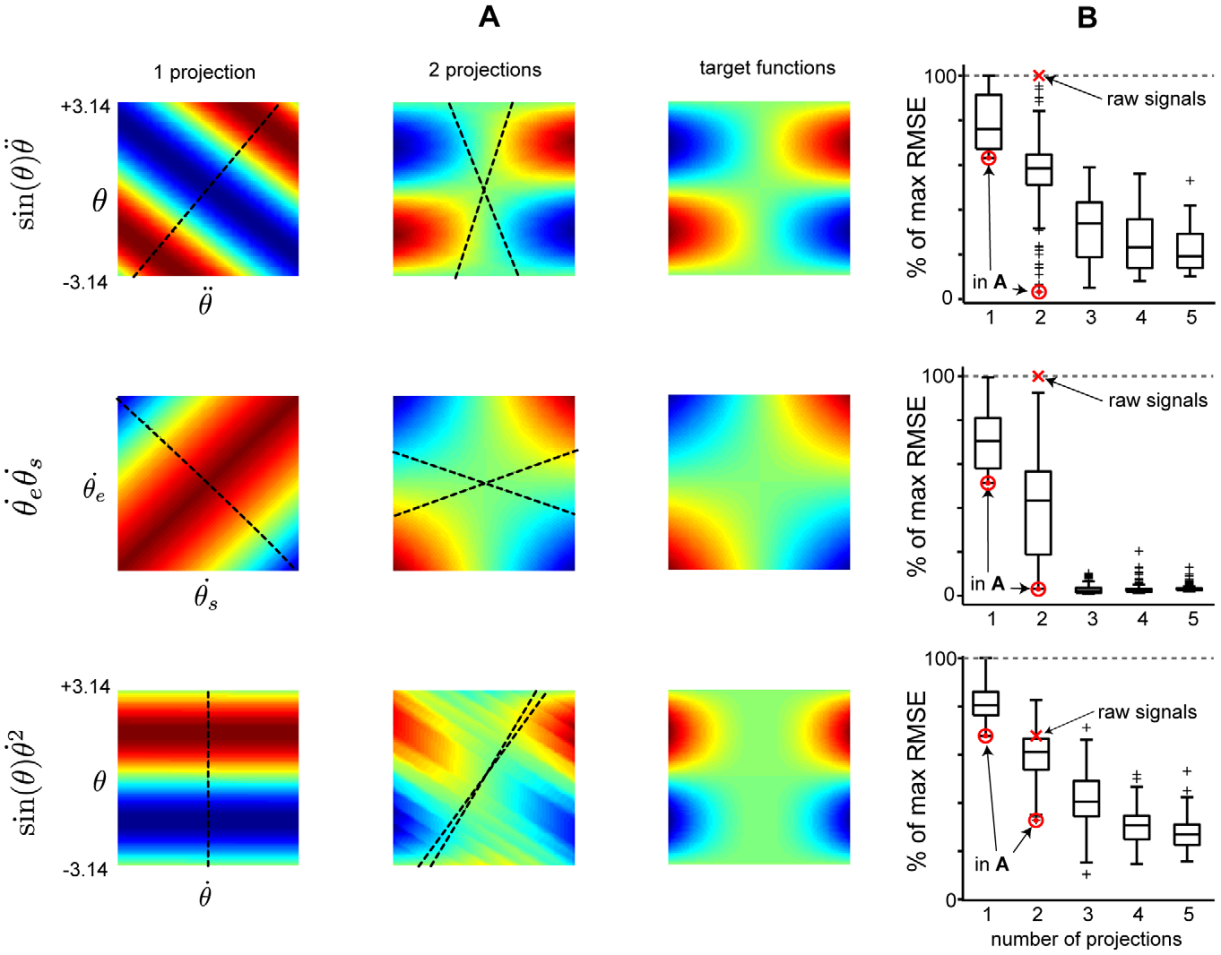
Approximation examples of basic non-linear functions. (A) Approximated surfaces using a single or two projections (left and middle columns, respectively) compared to the approximated or target function surface (right-hand column) (i.e RMSE = 0). The colors represent the height of the surface ranging from negative values (blue) to positive values (red), comparable to the surface in Figure 2C. In each row, a different non-linear interaction retrieved from the terms within the inverse dynamics of the planar double joint arm in [79] is used. The illustrated projections had the lowest RMSE of 100 tested projections, each tested projection having a random direction. The actual RMSE values can be found in (B). The approximated surfaces also display the actual projections used as dashed lines above the surfaces. The value of the elbow angle variable, 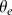 range between 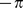 and 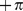, to capture an entire period of the sin function that is approximated. (B) RMSE of approximations of three two-dimensional non-linear terms in A. The approximations where constructed using random projection directions and a total of 60 GrCs. 100 approximations where constructed for each box. The mean RMSE is shown by the center line of the box, the boxes themselves extend to the 25th and 75th quartile and the whiskers extends to the most extreme RMSE not considered to be outliers, which are instead shown as black crosses. The red markers with an arrow from “raw signal” show the RMSE of approximations using the raw signals as projection directions, i.e. without recombination of inputs and those with an arrow from “in A” show the RMSE of the approximations shown in (A).

We explored how well non-linear functions could be captured using a limited number of projections, each represented in a limited number of GrCs, in a model of the SCT/SRCT systems and the cerebellar cortex to which the constraints described above applied. The functions that the model was assigned to approximate were a couple of central functions for monitoring intersegment dependencies in a multi-segmented limb (see ‘Application’), i.e. functions that have to be monitored somewhere in a system designed to achieve coordination control. Given what is known of the SCT and SRCT synaptic inputs and signals (see preceding sections), the signals necessary for approximating such functions might well be generated in these systems. Figure 4A illustrate how well the approximations created with the model in Eq. (4) could capture the non-linearities in Eq. (5) using a limited number of GrCs and a limited number of projections. The approximations with the lowest RMSE (relative to the actual function) using 60 GrCs along 1 and 2 projections, respectively, are compared to the actual functions (Figure 4A, right column). As it is hard to illustrate approximations with three regressors, the three-dimensional term 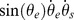, is exchanged with 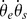 together with 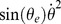 in Figure 4A. The colors illustrate the shape of the surface in a similar fashion as in Figure 2B, but in contrast to Fig. 2B the projections are not perpendicular to each other as in the naïve case with raw inputs, but indicated by the broken lines. Due to the symmetry of 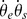, approximations with equal RMSEs can be obtained by rotating the projections 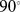 making them close to equal to the projections used for the optimal approximation of 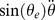. The best found approximation of 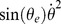 using two projections deviates with a considerably higher RMSE and the projections nearly overlap.

In a next step, we tested the effects of increasing the number of projections available to the system beyond two projections. Figure 4B illustrates that on average, using random projections, RMSE values improved substantially when moving from two to three projections. Notably, the approximation of 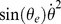, which was hard to approximate using two projections, could now be approximated with much better RMSE values. Figure 4B also indicates the RMSE values obtained for non-recombined raw signals (only two projections, corresponding to the two axes in the panels of Figure 4A), which generally lead to poorer results. Figure 4B in addition indicates separately the projections leading to the lowest RMSE values (i.e. the projections illustrated as dashed lines in Fig. 4A). This illustrates that out of a set of random projections, it is generally possible to find close to optimal recombinations (or, rather, ‘good enough’ recombinations, see Discussion).

In mathematical terms, already the relatively simple double joint arm gives rise to several functions that cannot be approximated using raw (i.e. non-recombined) inputs to the GrCs. E.g. for 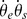 and 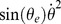, both the worst case using a single projection and the approximations using the raw inputs reached 100% of the maximum RMSE, i.e. a flat function surface equivalent to 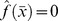. The average and worst case RMSE of the approximations was substantially improved as the number of available projections was increased (Figure 4B). The best obtained RMSE did however stay relatively constant as the number of projections was increased to two or more.

Concerning 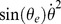, the approximation using both raw signals had no better RMSE than the approximation using a single ideal projection of both signals. Again, when the number of projections in the system is increased, the average and worst case RMSE was substantially reduced also in this case. At the same time, the best obtained RMSE did not improve as the number of projections was increased to three or more.

Common for all three functions was that low RMSEs could be obtained with only two or three projections (Figure 4B) and that the average RMSE of the approximations could be reduced by a rather modest increase in the number of projections, especially approximating 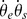. The above analysis indicates that recombinations of sensorimotor signals describing the non-linear dependencies between limb segments is necessary at the level of the cerebellar granule layer in order to create approximations of the functions in Eq. (55). At the same time, several GrCs can receive the same MF input without impairing the quality of the approximation in any significant way, allowing the recombination to take place already prior to the granule layer.

**Figure 5:**
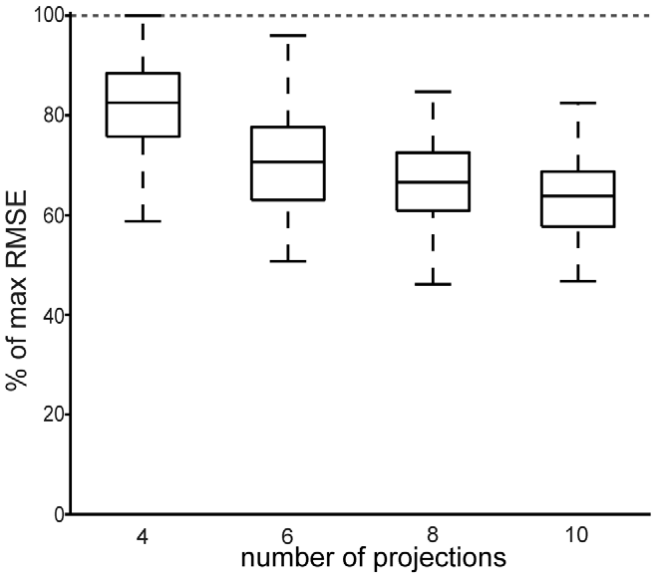
Three-dimensional approximations with random projections. Similar to the approximations in Figure 4B, but the approximated function is instead the three-dimensional term from Eq. (5), approximated over a three-dimensional grid. As in Figure 4, 60 GrCs were used and 100 approximations with random projections were constructed for each of the boxes. The mean RMSE is shown by the center line of the box, the boxes themselves extend to the 25th and 75th quartile and the whiskers extends to the most extreme RMSE.

**Figure 6:**
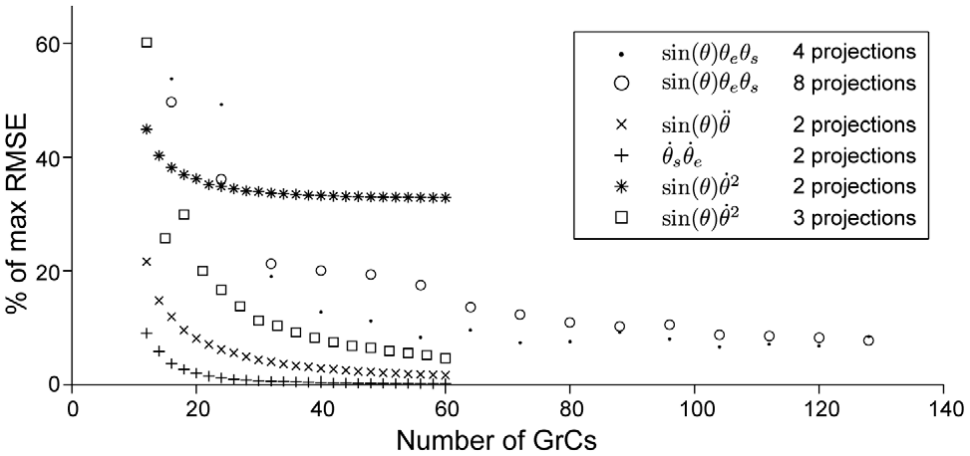
Approximation error when the number of granule cells is increased. The figure shows how the RMSE is reduced when the number of GrCs is increased as the functions in the figure legend were approximated using the specified number of projections. To search for the optimal approximations, the approximation directions were also optimized along with the GrC to PC weights. The first equation 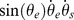, is three-dimensional and was approximated using both 4 and 8 projections, while the others are two-dimensional. Note that the last equation 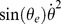 was approximated both using 2 and 3 projections to investigate the relatively large differences found using random projections (see Figure 4B).

While it was possible to find projections trough random selection that yielded good approximations of the 2-dimensional terms, the same is not true for the three-dimensional term 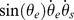 (see Figure 5). It should be possible to find approximations that reach the same RMSEs as with its two-dimensional counterpart 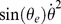 (see Figure 4B), but the constructed approximations indicate that random selection of projections is not sufficient to reach the same low RMSEs, even with a relatively high number of projections. However, by also including the projection directions to the approximation algorithm to search for the optimal projections, three-dimensional approximations with RMSEs comparable with their two-dimensional counterpart were found, even with a relatively low number of projections (see Figure 6). These observations indicate that the higher the number of dimensions, the worse the random projections would work. Hence, in particular for high-dimensional inputs, it would be highly inefficient for the spinocerebellar systems, and the cerebellar granule layer, to rely on random projections.

It should be noted that the algorithm did not converge by its own measure when Nelder-Mead was used, but had to be limited to a maximum number of iterations. Also, the algorithm did converge on local minima solutions and had to be restarted around the found minima to reach the solutions that are presented in Figure 6. There is no formal guarantee that the found solutions lie close to the global optima, and should be considered as upper bounds as better solutions could exist.

It is also interesting to note that the approximations using optimized projections had close to the same RMSE as the most accurate approximations created with random projections. By comparing the RMSEs of the 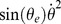 approximations using two and three projections in Figure 6, it is also evident that it is not enough to increase the number of GrCs in the approximation using two projections to reach the RMSE of the approximation obtained using three projections. However, as long as the number of projections is sufficient, additional projections does not seem to increase the accuracy as can be seen comparing the RMSEs of the 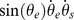 approximations using 4 and 8 projections in Figure 6. Altogether, these findings (i.e. from Figures 4–6) indicate that while it was possible to construct approximations of all the non-linear terms in Eq. (5) with small RMSEs, the properties of the function that is approximated determines how many projections that are necessary and how they should be selected.

## Discussion

Recombination of sensory and motor command signals in the spinocerebellar systems is likely a crucial circuitry feature to allow the cerebellum to be able to perform a variety of motor control functions such as coordination. Here we made a theoretical analysis of how this information can be used, based on some novel constraints coming out of *in vivo* analyses of cerebellar GrC function and the properties of the spinocerebellar systems. Important conclusions are:

1. Essential recombinations of sensory and motor signals are present already in the MF input as it enters the cerebellum. This may be a main role of the SCT/SRT systems.
2. There is a large number of GrCs sampling MF signals from functionally equivalent inputs, but they differentiate the signal by having different thresholds and by sampling input from sensors with somewhat different sensitivity. This is a necessary property for the PCs to be able to adequately approximate functions describing the states of the body and the nervous system.
3. The number of potential projections within the high-dimensional sensorimotor information space is essentially infinite, implying that the GrCs and the SCT/SRCT systems must be highly selective with respect to which recombinations are represented.
4. These considerations has the surprising consequence that the number of GrCs, despite that these are by far the most numerous neuronal element within the brain [75], is a numerical bottle-neck for how well the cerebellum can approximate the properties of our bodies and object-body interactions.

### Assumptions and constraints – when does the model not hold?

A main focus of our hypothesis is that the spinal circuitry makes a number of sensorimotor functions, useful in the task of coordination, available to the cerebellar cortex through its MF projections via the SCT/SRCT systems. A second component of the hypothesis is how this information could be received by the cerebellar granule layer. Here, our hypothesis and the constructed model rests on a number of experimental observations *in vivo*, which suggest that rate coding is the predominant form of coding in MFs, that GrCs receive input from functionally similar MFs and that the dominant mode of control of excitability in GrCs is through tonic inhibition, presumably mainly through Golgi cells. One of our main assumptions is that these observations apply. On the view of the cerebellar cortex, the hypothesis extends the adaptive filter hypothesis [40], [82] and addresses the some of the questions raised in a recent review [40] related to the role of the granule layer and the MF signaling that follow from the described in vivo findings. Naturally, in cases where the MF systems would operate primarily through spike time coding and where inhibitory synaptic inputs from Golgi cells to GrCs primarily operates through fast, phasic IPSPs, very different functional interpretations of the function of the granule layer would apply. Under these circumstances other functional models of the granule layer, similar to those presented in other reviews and models [83], [84], would become more likely. At present, however, support for our assumptions from in vivo recordings seems relatively strong. All recordings of MFs in awake animals during natural behavior seem to support the interpretation that MFs operate with rate coding, in particular for the spinocerebellar systems and within the limb control zones that we are considering here [46], [49]–[52], [85], [86] but also for MFs in the oculomotor controlling regions of the cerebellum [48] and quite possibly also for vestibulocerebellum [47]. Also Golgi cells seem to follow the rate coding principle in relation to controlled movement [48], [47]. Similarly, although intracellular recordings in GrCs in vivo are rare, studies of their inhibitory responses in vivo are rarer still, but those available indicate that a prominent tonic inhibition is existent [21], that in the adult animal fast IPSPs are difficult to detect [29], whereas tonic inhibition under Golgi cell control is demonstrable [88], and that even in the juvenile animal (which is remarkable considering that full maturation of the Goc-grc inhibition does not occur until adulthood, see Introduction) more than 98% of the inhibitory charge is carried by tonic inhibition [89]. Hence, although the predominance of tonic inhibition in the Golgi cell to GrC inhibition is still controversial, so far all in vivo studies available support this idea.

Notably, another controversy with respect to GrC function, which revolves around the question whether single MFs on high-frequency repetitive activation can cause the GrC to fire or not [40], [90], does not affect the functional view presented here. As long as the MF – GrC transmission has a threshold this functional view would not change. In fact, a varying threshold across the population of GrCs, which is beneficial for our present model, could possibly in some cases result in that single MFs would have such heavy influence on GrC firing also *in vivo*, although this remains to be shown [40].

Note that as long as the inhibition exerted on GrCs is predominantly tonic, the exact timing of Golgi cell spikes, although regulated through an intricate set of mechanisms [91], has a comparably small impact in this model.

### Possible functional roles of Golgi cells in our model

In the present hypothesis, Golgi cells or GABAergic control of GrCs have the important function of diversifying the GrC thresholds to maximize the ability of the PCs to make piecewise-linear approximations of any function that is useful for fulfilling their roles in motor control. Although the source of GABA that sustain the tonic inhibition of GrCs is somewhat controversial, and may include for example glial sources [92], assuming that there is a contribution from GABA released by Golgi cells has some interesting consequences. By regulating Golgi cell activity, it might then be possible to give the same GrC different thresholds in different contexts. This would depend on the fact that MFs representing a given input have focal terminations [35], [37], [53], which should create clusters of GrCs with functionally similar inputs, and that Golgi cells inhibit primarily local clusters of GrCs [12], [93]. Hence, by modulating the Golgi cell spike firing to different depths [88], which would result in a modulated tonic inhibition [87], the cerebellar cortex could assign different specific roles to the same GrC in different contexts, even though the input it receives from the MFs are still coding for the same function. Exploring these issues, and the mechanisms of how the cerebellar cortical circuitry could regulate the thresholds of specific GrC clusters in different contexts, are important outstanding issues for future cerebellar research.

### Crucial role of the spinal circuitry in presenting useful sensorimotor functions

Although it is known that the spinal neurons of origin of the SCT and SRCT pathways integrate sensory feedback with descending motor command signals, the idea that this integration is used to provide the cerebellum with sensorimotor functions or projections into sensorimotor space is a fundamental assumption in the current hypothesis. This assumption has not been extensively explored, but there is certainly data that is consistent with this idea [94], [95].

As argued in the Model and Hypothesis section, in relation to the potential number of projections that could be formed within the complex sensorimotor space of our bodies, the cerebellum and the number of GrCs that are available poses a severe limit on the number of projections that can be represented. It follows that the selection of these projections is an important step for the organism so that the most useful calculations, relative to the sensorimotor apparatus available, is made available to the cerebellum.

How these projections are established and configured are crucial outstanding issues, but a number of observations suggest that the spinal cord is an appropriate place to take the decisions of which projections that are relevant. Simulations using a realistic network structure and local sensory feedback patterns have shown that the spinal circuitry does provide a number of useful sensorimotor functions that can be played on by using motor commands [96]. The connectivity of the circuitry is established through plasticity processes whose outcome depends on the configuration of the sensorimotor apparatus [97] and the existence of spontaneous movements [98]. In other words, the development of the spinal circuitry is adapted to the development of the sensorimotor apparatus and the correspondence between movement and sensory feedback. It is likely that individual neurons of the spinal circuitry during the development ‘finds’ appropriate combinations of sensors and motor command signals, associations that are helpful for brain movement control by providing such assistive sensorimotor functions. The close correspondence between the sensor signals and motor command signals in the individual neurons could be a means for the spinocerebellar system to avoid superfluous recombinations (which the system can certainly not afford, see argumentation under ‘Limited number of GrCs’) and that ‘good enough’ recombinations (close to optimal) might be formed with a high probability. Describing the processes that establish the precise patterns of recombinations made available by individual spinal interneurons could be a particular intriguing example of advanced learning processes within the central nervous system and is another crucial outstanding issue for future research.
